# Importance of immature platelet fraction as predictor of immune thrombocytopenic purpura

**DOI:** 10.12669/pjms.323.9456

**Published:** 2016

**Authors:** Arshi Naz, Samina Naz Mukry, Mahwish Rauf Shaikh, Ali Raza Bukhari, Tahir Sultan Shamsi

**Affiliations:** 1 Arshi Naz, Ph.D. National Institute of Blood Disease and Bone Marrow Transplantation, Karachi, Pakistan; 2 Samina Naz Mukry, Ph.D. National Institute of Blood Disease and Bone Marrow Transplantation, Karachi, Pakistan; 3 Mahwish Rauf Shaikh, M.Sc. Jinnah University for Women, Karachi, Pakistan; 4 Ali Raza Bukhari, M.Sc. National Institute of Blood Disease and Bone Marrow Transplantation, Karachi, Pakistan; 5 Tahir Sultan Shamsi, FRCPath. National Institute of Blood Disease and Bone Marrow Transplantation, Karachi, Pakistan

**Keywords:** Immune thrombocytopenic purpura (ITP), immature platelet fraction (IPF)

## Abstract

**Background and Objective:**

Immune thrombocytopenic purpura (ITP) is a clinical syndrome in which a decreased number of circulating platelets (thrombocytopenia) manifests as a bleeding tendency, easy bruising (purpura) or extravasation of blood from capillaries into skin and mucous membranes (petechiae). The diagnosis of ITP can be made clinically on the basis of symptoms, we need to see if ITP can be confirmed in patients by quantification of residual RNA containing immature platelets (megakaryocytic mass) or immature platelets fraction (IPF) using automated hematology analyzers (Sysmex XE-2100).

**Methods:**

In order to check the efficacy of IPF% parameter of Sysmex XE-2100 a total of 231 patients of thrombocytopenia were included in this study. Complete blood count (CBC) was estimated. The data was statistically analyzed by SPSS version 17.

**Results:**

About 62 patients were diagnosed as ITP and 169 patients were diagnosed as non ITP on the basis of clinical history. The mean IPF % value of ITP patients was 16.39% and the IPF % value of Non ITP patients was ~7.69% respectively. There was no significant difference in IPF% values with respect to time between sampling and acquisition of complete blood count. The diagnostic sensitivity of IPF% as biomarker for ITP and non-ITP was 85.71% (95%CI: 84.04% to 85.96%) and 41.76% (95% CI: 39.87% to 43.65%).

**Conclusion:**

The mean IPF % value by Sysmex XE-2100 can be used to predict ITP.

## INTRODUCTION

The platelets are the key player in controlling bleeding problems. Abnormally low/ decreased number of circulating mature platelets (thrombocytopenia) may lead to excessive bleeding, easy bruising (purpura), or extravasations of blood from capillaries into skin and mucous membranes (petechiae).^1^ This condition was previously termed as idiopathic thrombocytopenia purpura, now it is well known that in most of the cases cause is not idiopathic but autoimmune with antibodies against platelets being detected in patients and associated with high megakaryocytic mass in bone marrow. The present terminology for this condition is immune-thrombocytopenia purpura (ITP).^1^

Major cause of thrombocytopenia in ITP is accelerated platelet destruction and impaired platelet production. Exact etiology is unknown. The global incidence of acute ITP in children is between 1.9 and 6.4 per 10^5^ children/year; for adults the estimate is 3.3 per 10^5^ adults/year.^2^

Following an episode of acute blood loss reticulated platelets (RP) containing remnants of RNA are generally observed. In case of ITP the number of such platelets is markedly high due to excessive peripheral platelet destruction.^3^ Previously, RNA condensations in platelets were observed by microscope, similar to immature red blood cells after staining of the reticulum.^4^ Several flow-cytometric methods have been described in the past 20 years. Most of these are not standardized, time consuming and requires skilled laboratory staff.^5^ Besides flow-cytometric quantification of RP the inexpensive test for rapid assessment of immature platelet function (IPF) by automated hematology analyzers, may be useful to evaluate patients with thrombocytopenia. Testing on ITP patients demonstrated consistent IPF elevations, typically >10%, with normal or near normal results in patients with marrow suppression.^4^ Borderline elevations may occur with liver cirrhosis, presumably due to the mixed etiology of low platelet count in these cases involving both increased platelet production and decreased hepatic thrombopoietin production.^6^ Present study was done to observe the importance of immature platelet fraction for the diagnosis of ITP in patients.

## METHODS

This study was conducted at National Institute of Blood Diseases & Bone Marrow Transplantation (NIBD), Karachi, from September 2012 to January 2013. About 227 patients with different age groups of both genders diagnosed as having thrombocytopenia were included. Patients with other causes of thrombocytopenia (inherited thrombocytopenias), bone marrow failure syndromes, lymphoproliferative disorders, myeloproliferative disorders, thrombotic microangiopathies, megaloblastic anaemia and pseudothrombocytopenia were carefully excluded according to the findings in history, physical examination and investigations.

The diagnosis of the patient groups included ITP with platelet counts of less than 150×10^9^/L. ITP was diagnosed on the basis of the patient’s medical history, isolated thrombocytopenia without other underlying diseases, and additional laboratory tests, including anti-platelet antibody tests and bone marrow examination, where necessary. The ITP positive cases were confirmed by morphological examination of stained peripheral blood smears. For the determination of diagnostic efficacy of the biomarker (IPF%) about 94 healthy individuals of either sex were also included as control group

### Sample collection

Peripheral blood samples (3 mL) collected in K_2_EDTA (Beckton Dickinson, Franklin Lakes, NJ, USA) were analyzed for IPF% and all routine (full blood count) parameters, including platelet counts. All samples were kept at room temperature and were analyzed within 8 hrs after collection.

### Measurement of IPF%

The IPF% was measured using Sysmex XE-2100 (TOA Medical Electronics, Kobe, Japan). The IPF% results were available at the same time as the full blood counts were done. The IPF% of each individual sample was acquired twice right after collection (within 15 minutes.) and results were recorded as mean of two independent runs. The platelets count and mean platelets volume (MPV) were also recorded as comparative parameters. Total 40 samples; 20 each from confirmed ITP cases and non-ITP cases were re-analysed after 7.5 hrs of collection to observe any change in IPF% with time.

### Statistical analysis

The data was statistically analyzed by SPSS version 17. The descriptive parameters were described in terms of mean±standard deviation or median whereas inferential parameters for ITP, non-ITP and normal healthy individuals were statistically analyzed by Chi square test at significance level 95%.

## RESULTS

**Fig.1:**
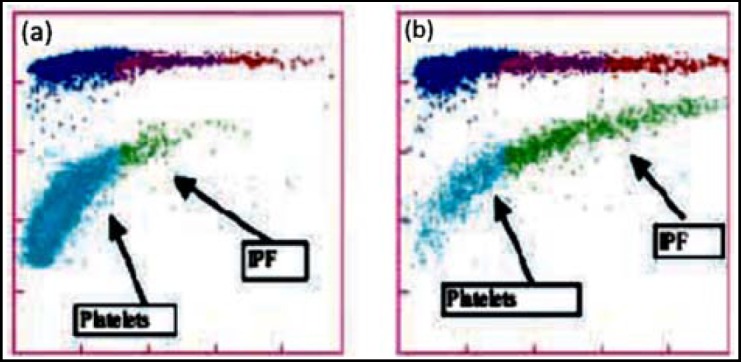
Optical platelet scattergrams from a healthy individual with a normal IPF (a) and a patient with a high IPF (b). Mature platelets appear as blue dots, green dots represent the IPF with increased cell volume and higher fluorescence intensity compared to mature platelets.

A total of 62 patients were diagnosed as ITP and 169 patients were diagnosed as non ITP on the basis of clinical history and laboratory tests. The median age was 23 years, 32 years and 33 years for the ITP and non-ITP patients and healthy controls respectively (Table-I). There were 32 female ITP patients and 28 were males. The mean platelet count of ITP patients was 61.10±52.58x10^3^/ul and platelet count of non ITP patients was 54.62±39.64x10^3^/ul summarized in Table I and II. The normal range for IPF% is 1-7% while greater than 7% is considered as abnormally raised (Fig.1). As observed the mean IPF% for ITP patients was 16.39% while for non-ITP patients it was 7.69% i.e. within normal range The MPV% and IPF% were statistically significant with a p value of <0.0001. There was no significant difference between the IPF% values of patients recorded within 15 minutes of collection and after 7 ½ hours of collection. The mean IPF % for the selected ITP and non-ITP patients after 15 minutes and 7 ½ hours of collection was 20.93%±0.22 and 6.43%±0.24 respectively. The diagnostic sensitivity of IPF% as predictive bio-marker was found to be variable while the specificity was 70.21% (95% CI: 68.85% to 71.57%) in both cases. The sensitivity for ITP and non-ITP was 85.71% and 41.76% respectively (Table-III).

**Table-I:** Demographic characteristics of study subjects.

	*N (Male/ Female)*	*Age years median*	Hemoglobin^*^ g/dl Mean±SD	Platelets^*^ 10^3^/ul Mean±SD	RBCs^*^ 10^6^/ul Mean±SD	WBCs^*^ 10^3^/ul Mean±SD
ITP	62 (30/32)	23	11.99±2.10	61.10±52.58	4.49±0.63	9.08±4.27
Non-ITP	169 (100/69)	32	9.01±3.10	54.62±39.64	3.23±1.10	23.53±65.54
Normal	94 (52/42)	33	12.68±1.11	207.08±32.00	5.02±0.49	8.49±1.70

* Readings were recorded as mean of two independent runs on Sysmex XE-2100.

**Table-II:** Platelets parameters in ITP, Non-ITP patients and normal individuals.

	*Platelets 10^3^/ul*	MPV*ƒL	*IPF>%*
ITP	61.10±52.58	10.84±1.94	16.39±11.15
Non-ITP	54.62±39.64	10.65±1.5	7.69±6.09
Range of normal	164-237.08	9.6-11.12	1.1-17.8
p	0.08	<0.0001	<0.0001

p-value was determined by Chi square test; values ≤0.05 were considered as significant

* Mean of available MPV values. No MPV value was calculated in 38 ITP (75.80%) and 110 (65.08%) non ITP cases due to instrumental limitation.

**Table-III:** Comparative efficacy of IPF% as a biomarker for preliminary diagnosis of ITP and other Non-ITP thrombocytopenias.

	Positive Predictive Value (95% CI)*	Negative Predictive Value (95% CI)*	Sensitivity (95% CI)*	Specificity (95% CI)*
ITP	77.14% (72.48% to 81.80%)	78.57% (77.43% to 79.71%)	85.71% (84.04% to 85.96%)	70.21% (68.85% to 71.57%)
Non-ITP	81.6% (79.33% to 83.87%)	25% (22.85% to 27.15%)	41.76% (39.87% to 43.65%)	70.21% (68.85% to 71.57%)

* 95% confidence interval (CI)

## DISCUSSION

ITP may be diagnosed on the basis of clinical findings but laboratory confirmation is often required.^1^ Previously, microscopic examination of bone marrow aspirates was performed to confirm ITP. Since the bone marrow extraction itself is a painful procedure therefore later on flowcytometric assays were employed to jump to final diagnosis. But being time consuming and inconsistent nature of these tests limit their application.^7^ At present automated quantification of reticulated platelets has been used to discriminate between cases of severe thrombocytopenia and ITP. Published literature clearly showed that, under conditions of thrombocytopenia, the platelet RNA content correlated directly with megakaryocytic activity.^4^,^8^ The IPF% is a novel parameter available on the Sysmex XE-series hematology analyzers. It can be expressed as both IPF percent (IPF%) and absolute IPF count (AIPF#). IPF% can be used as a non-invasive parameter to monitor patient response, particularly when changing immunosuppressive therapy. The measurement of the IPF is simple and rapid. It depicts the bone marrow megakaryocytic activity and platelet lifespan. It may be used as a platelets recovery marker in hematopoietic stem cell transplant recipients.^9^ Studies suggest that this parameter may also be used as indirect biomarker of poor prognosis in myelodysplastic syndrome with karyotypic abnotmalities.^10^

It was observed that IPF% was higher or >7% in ITP than in the normal population, and correlates with bone marrow platelet production or thrombopoiesis. These observations were in line with other studies. 11-13 The sensitivity and specificity of IPF has previously been reported to be 91–96% and 67–100%, respectively.^14^ In the present study the sensitivity and specificity of IPF% as biomarker for ITP was found to be 85.71% and 70.21% (Table-III). Eighty five percent of total ITP patients had a raised IPF% whereas 96.15% of ITP patients with a total platelet count of <50x10^9^/l had a raised IPF%. There was significant inverse correlation of platelet count with IPF%; the lower the platelet counts, the higher the IPF%. The IPF% value reflected the severity of platelet destruction. With a poor sensitivity of about 41.76% for non-ITP thrombocytopenias IPF% may not be considered as a good biomarker for these cases. Further studies are indeed required to justify this conclusion.

The reproducibility of the method of duplicate analysis was good for most parameters. Beside IPF% the MPV is another parameter used to make inferences about platelets production in bone marrow or platelets destruction problem (Normal range: 7.5-11.5fL). Higher MPV values associated with increased platelets destruction is a common observation in inflammatory bowel disease, ITP and myeloproliferative diseases.^10^,^13^ It is a machine calculated ratio of platelets crit and platelets count multiplied by 1000. It was interesting to observe that in about 75.80% ITP and 65.08% non-ITP cases the Sysmex XE-2100 was unable to calculate MPV because of unreadable plateletcrit which might be taken as zero or out of range by the machine. The mean of available MPV for ITP, non-ITP and normal controls was found to be statistically significant (p value ≤ 0.0001). Thus despite being statistically significant; due to high number of missing values MPV cannot be taken as a diagnostic marker and might be one of the limitation of the present study. Since no significant difference in IPF% was observed with time of acquisition so this test may be performed at any point of time within 8 hours of sample collection.

## CONCLUSION

ITP is primarily a disease of increased peripheral platelet destruction, and this study demonstrates a fully automated, rapid method for the determination of the IPF, using the Sysmex XE-2100 equipped with upgraded software to analyze the highly fluorescent platelets. The results are available at the same time as the full blood count and the IPF is reproducible and stable in an EDTA sample stored at room temperature for at least 8 hrs. The IPF% provides a valuable diagnostic method to clearly establish the diagnosis of idiopathic (or immune) thrombocytopenic purpura in patients. Hence it can be emphasized that the IPF should become a standard routine parameter in the diagnosis and serial monitoring of the thrombocytopenic patient.
